# Hospital pharmacist discharge care is independently associated with reduced risk of readmissions for patients with chronic obstructive pulmonary disease: A propensity-matched cohort study

**DOI:** 10.1177/17151635211061141

**Published:** 2021-12-10

**Authors:** Joy Makari, Joseph Dagenais, Mina Tadrous, Sarah Jennings, Israa Rahmaan, Kaleen Hayes

**Affiliations:** Queensway Carleton Hospital, Ottawa; Queensway Carleton Hospital, Ottawa; Leslie Dan Faculty of Pharmacy, University of Toronto, Toronto; Women’s College Hospital, Toronto, Ontario; Queensway Carleton Hospital, Ottawa; Queensway Carleton Hospital, Ottawa; Dalla Lana School of Public Health, University of Toronto, Toronto

## Introduction

Chronic obstructive pulmonary disease (COPD) is a leading cause for hospitalization and death globally,^1^ and the number of people affected by this disease is expected to more than double every 10 years.^2^ Today, COPD places excessive stress on the health care system, as hospitalization rates exceed those of any other chronic disease, including angina, heart failure, diabetes or hypertension,^3^ and direct costs attributable to COPD in the past 10 years in Canada have exceeded $1.5 billion.^4^ One in 5 patients hospitalized for COPD will require readmission within 30 days.^1^ Hospital readmission is associated with a decline in lung function, lower quality of life and increased mortality risk for patients with COPD,^5^ yet up to 50% of readmissions are potentially preventable by health care provider interventions.^5^ Evidence-based, multidisciplinary interventions are needed to address the growing public health issue of long-term COPD management and hospital readmissions.

To address this issue, the Queensway Carleton Hospital instituted a quality assurance (QA) program for patients admitted with COPD. This program included pharmacist care where possible, which may allow us to evaluate the incremental effect of pharmacist care on top of the quality assurance (QA) program. Therefore, the primary objective of this study was to evaluate the impact of hospital pharmacist discharge care, including counselling, for COPD patients on 30-day readmission and emergency department (ED) visits when delivered as part of a hospital-wide QA program.

## Methods

### Study design

Using a retrospective cohort study design, we measured the effects of hospital pharmacist discharge care on 30-day ED visits and 30-day hospital readmissions for patients discharged between November 1, 2016, and March 15, 2020. As part of a hospital-wide, quality improvement initiative at Queensway Carleton Hospital, a 264-bed acute-care hospital in Ottawa, several interventions were made to usual care to reduce short-term health care utilization among patients with COPD beginning in 2016. For every patient with COPD being discharged, the following standard interventions were applied: nursing checklists, telephone follow-up calls and a follow-up visit in the COPD nursing clinic, as required. Inpatient nurses provided initial inhaler teaching and reinforced inhaler technique throughout the duration of hospitalization. Charge nurses conducted a postdischarge follow-up phone call within 48 hours of discharge. The COPD clinic nurse reinforced education for patients on inhaler technique at follow-up appointments as well as through remote video consultation when needed. In addition to these full-time interventions, a part-time hospital pharmacist medication discharge care initiative (2 to 5 days per week as dictated by department needs) was implemented (details of the intervention are outlined in Figure 2). All adult patients 35 years or older admitted to acute care with COPD requiring treatment beyond maintenance (identified at admission through pharmacist chart review) were eligible for hospital pharmacist discharge care. Both inpatient nurses as well as the COPD clinic nurse aided in identifying eligible patients for hospital pharmacist discharge care. Palliative or alternate level-of-care^3^ patients were excluded.

Eligible patients were provided with discharge counselling by a COPD pharmacist when the pharmacist was available, the pharmacist received adequate notice of the discharge (≥30 minutes prior) and the patient accepted counselling. Patients declining counselling or those discharged when the pharmacist was unavailable received usual care (i.e., all other discharge interventions). Hospital pharmacist discharge care included a medication review and reconciliation, a medication list outlining any changes to pharmacotherapy regimens, medication and nonpharmacologic (e.g., smoking cessation) counselling, inhaler teaching and instructions regarding when the patient should seek medical attention postdischarge. In addition, the pharmacist provided a follow-up call 1 to 2 weeks postdischarge. At the point of discharge or during telephone follow-up, pharmacists also offered patients referral to a local community-based paramedic program, enabling nonemergent postdischarge in-home wellness checks by a paramedic. Patient demographics, clinical information, counselling status, death data and acute-care outcomes were captured via chart review of our hospital’s electronic health record (EHR) system. All patients who experienced a readmission also had an ED visit.

### Analysis

We evaluated outcomes in those patients who received the pharmacist COPD intervention compared to those who did not. To account for any differences in patient characteristics between groups, we used a logistic regression model with the following variables: sex, smoking status, home oxygen use, length of current hospitalization and comorbidities (e.g., asthma, renal impairment, cardiovascular disease, diabetes mellitus). We matched noncounselled patients to counselled patients at a 1:1 ratio using a greedy matching algorithm without replacement within a 0.1 caliper of the logit propensity score.^6^ Characteristics of matched patients were compared using standardized mean differences (SMDs), with an SMD of 0.10 indicating no meaningful difference between groups.^7^

We used logistic regression models to estimate odds ratios (ORs) and 95% confidence intervals (CIs) for the effect of hospital pharmacist discharge care on outcomes. For any statistically significant effect in logistic regression models, we used a log-linear regression model to calculate a risk difference (RD) and 95% CI. As a sensitivity analysis, any variable that remained imbalanced after matching was included in the regression models to determine if adjustment changed counselling’s effect (>10%). We obtained approval from the Queensway Carleton Hospital Research Ethics Board to conduct this study (Study Approval 20-03). All analyses were conducted using SAS version 9.4 (SAS Institute, Cary, North Carolina).

## Results

**Figure 1:**
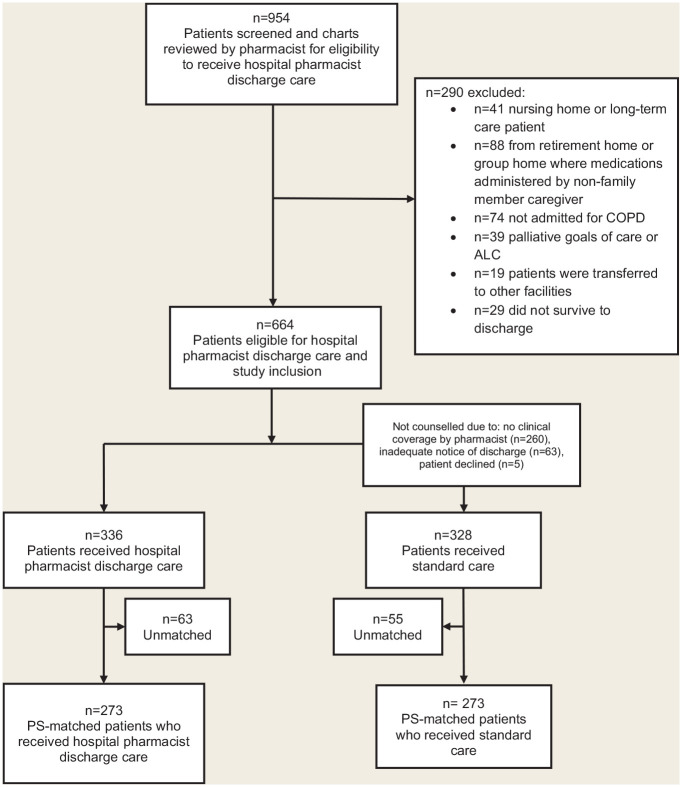
Study flow diagram ALC, alternate level of care status—a patient occupying a bed in a facility who does not require the intensity of resources/services provided in that care setting (acute, chronic or complex continuing care, mental health or rehabilitation);^3^ COPD, chronic obstructive pulmonary disease; PS, propensity score.

Pharmacists screened 954 patients to receive hospital pharmacist discharge care over 40 months, and 290 patients were excluded (Figure 1). Among the 664 eligible patients, 336 received hospital pharmacist discharge care and 328 did not, with 5 of these patients declining pharmacist counselling. In all, 273 patients who did not receive hospital pharmacist discharge care were propensity score matched to 273 patients who received hospital pharmacist discharge care, for a total of 546 patients in the final analysis. Characteristics were well balanced between patients receiving hospital pharmacist discharge care and those who did not, except body mass index (BMI) category (SMD 0.14, Table 1). Characteristics of patients whowere not matched are presented in Appendix 1 (available at www.cpjournal.ca).

**Table 1:** Characteristics of propensity score–matched patients (*N* = 546)

Characteristic	Hospital pharmacist discharge care (*n* = 273)	Standard care (*n* = 273)	Standardized mean difference
Age (mean, SD)	74.7 (9.5)	74.6 (10.8)	0.00
Male	95 (34.8)	107 (39.2)	0.09
Number of hospitalizations in past 6 months (mean, SD)	0.50 (0.80)	0.60 (1.0)	0.09
Length of hospitalization			
≤7 days	161 (59.0)	166 (60.8)	0.04
>7 days	112 (41.0)	107 (39.2)	0.04
COPD severity*			
Mild or moderate (FEV1 ≥50% predicted)	59 (21.6)	56 (20.5)	0.04
Severe or very severe (FEV1 less than 50% predicted)	135 (49.5)	140 (51.3)	0.04
Missing/unknown	79 (28.9)	77 28.21	0.04
Patient-reported smoking status—yes	86 (31.5)	85 (31.1)	0.00
Home oxygen use	89 (32.6)	85 (31.1)	0.03
Coronary artery disease	55 (20.2)	53 (19.4)	0.02
eGFR 30-60 mL/min	60 (22.0)	56 (20.5)	0.03
eGFR <30 mL/min	12 (4.4)	12 (4.4)	0.00
Heart failure	39 (14.3)	40 (14.7)	0.01
Hypertension	162 (59.3)	163 (59.7)	0.00
Dementia or cognitive impairment	33 (12.1)	35 (12.8)	0.02
Diabetes mellitus	55 (20.2)	62 (22.7)	0.06
Atrial fibrillation or aflutter	55 (20.2)	52 (19.1)	0.03
Asthma	21 (7.7)	28 (10.3)	0.09
Body mass index category*			0.14
Underweight	52 (19.1)	7 (2.6)	
Normal	81 (29.7)	41 (15.0)	
Overweight	56 (20.5)	84 (30.8)	
Obese (class I, II or III)	71 (26.0)	68 (24.9)	
Missing/unknown	13 (4.8)	73 (26.7)	
Emergency department visit within 30 days (outcome)	61 (22.3)	68 (24.9)	
Rehospitalization/readmission within 30 days (outcome)	31 (11.4)	50 (18.3)	

Values are presented as number (%) unless otherwise indicated.

COPD, chronic obstructive pulmonary disease; eGFR, estimated glomerular filtration rate; FEV1, forced expiratory volume in 1 second.

* Not included in the propensity score model due to >5% patients missing information.

**Figure 2:**
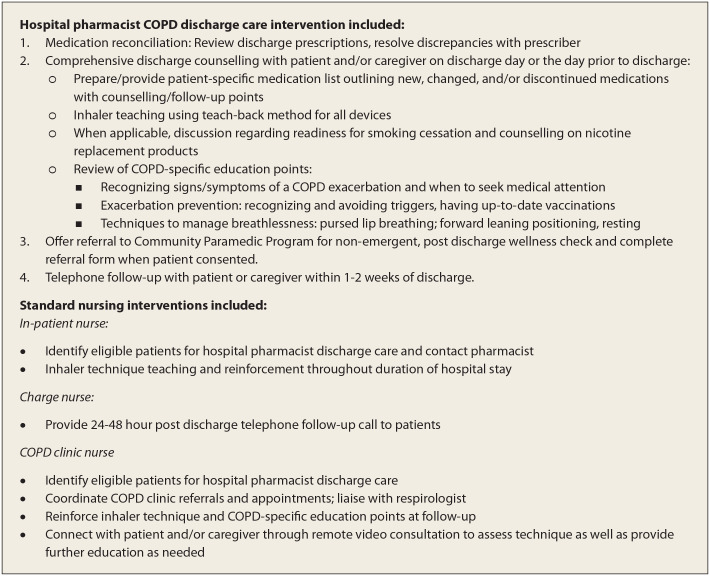
Overview of hospital pharmacist discharge care intervention

After discharge, 129 patients (24%) experienced an ED visit and 81 patients (15%) experienced a hospital readmission within 30 days. Hospital pharmacist discharge care was associated with a significant decrease in readmissions (OR, 0.57; 95% CI, 0.35-0.93; *p* = 0.02), Table 2. This effect translated into a 7% decreased absolute risk of 30-day readmissions for patients who received discharge care from hospital pharmacists vs patients who did not (95% CI, 1.0%-12.9% decreased risk). Hospital pharmacist discharge care did not have a significant effect on 30-day ED visits (OR, 0.87; 95% CI, 0.58-1.29; *p* = 0.48). For the sensitivity analysis, we included BMI category in the logistic regression model for the 460 patients with this information available and found no substantial change in results for readmissions (OR, 0.60; 95% CI, 0.37-0.97).

**Table 2:** Effect of hospital pharmacist discharge care on 30-day readmissions and emergency department visits

Outcome	Hospital pharmacist discharge care (N = 273) N (%)	Standard care (N = 273) N (%)	Odds ratio (OR) 95% confidence interval (CI)	p-value
Emergency department visit within 30 days	61 (22.3)	68 (24.9)	OR 0.60 [95% CI 0.37 to 0.97]	P=0.48
Rehospitalization / readmission within 30 days	31 (11.4)	50 (18.3)	OR 0.57[95% CI 0.35 to 0.93]	P=0.02

## Discussion

We conducted a single-centre, propensity score–matched cohort study and found that hospital pharmacist discharge care, on top of a hospital discharge program for patients with COPD, was associated with a reduced risk of 30-day hospital readmissions independent of other interventions. The absolute risk reduction of 7% for readmissions translates to a 30-day number needed to treat of 15 patients receiving discharge care to prevent 1 readmission. While prior studies have demonstrated relative reductions in readmissions for patients with COPD receiving pharmacist counselling or pharmacist discharge care,^8-10^ this is the first study to use propensity score matching to examine the additive absolute effect of hospital pharmacist discharge care on top of other standardized care improvements. Hospital pharmacist discharge care had no effect on 30-day ED visits, perhaps because patients were counselled to seek medical attention at the first sign or symptom of an exacerbation. An increase in ED visits at earlier exacerbation stages among hospital pharmacist discharge care patients may have contributed to the observed reduction in readmissions.

Our findings are from a single Canadian hospital, potentially limiting generalizability, yet the rate of readmission among the usual-care group (18%) was similar to that of other populations.^4^ In addition, we were restricted to readmission data specific to our institution and may not have captured acute-care use in other systems. However, we expect that counselled patients would be more likely to seek care at our hospital, reducing the intervention’s observed effect. Although we employed propensity score matching using many prognostic characteristics, missing information such as socioeconomic factors may be related to readmission. Finally, randomization in future study designs will help confirm the causality of our results.

In summary, hospital pharmacist discharge care for patients with COPD appeared to reduce the odds of 30-day readmissions by 43%. Multicentre, randomized studies will determine whether these results are replicable and generalizable on a broader scale. ■

## Supplemental Material

sj-pdf-1-cph-10.1177_17151635211061141 – Supplemental material for Hospital pharmacist discharge care is independently associated with reduced risk of readmissions for patients with chronic obstructive pulmonary disease: A propensity-matched cohort study Click here for additional data file. Supplemental material, sj-pdf-1-cph-10.1177_17151635211061141 for Hospital pharmacist discharge care is independently associated with reduced risk of readmissions for patients with chronic obstructive pulmonary disease: A propensity-matched cohort study by Joy Makari, Joseph Dagenais, Mina Tadrous, Sarah Jennings, Israa Rahmaan and Kaleen Hayes in Canadian Pharmacists Journal / Revue des Pharmaciens du Canada
