# Supplementary material for: Hospital pharmacist discharge care is independently associated with reduced risk of readmissions for patients with chronic obstructive pulmonary disease: A propensity-matched cohort study
Source: Can Pharm J (Ott). 2021 Dec 10;155(2):101–6. doi: 10.1177/17151635211061141 (PMC8922223; doi:10.1177/17151635211061141)
Supplement: sj-pdf-1-cph-10.1177_17151635211061141 – Supplemental material for Hospital pharmacist discharge care is independently associated with reduced risk of readmissions for patients with chronic obstructive pulmonary disease: A propensity-matched cohort study [file sj-pdf-1-cph-10.1177_17151635211061141.pdf]

APPENDIX 1 Characteristics of non-matched patients (N=118)

Characteristic	Unmatched patients who received hospital pharmacist discharge care (N=63) N (%)	Unmatched patients who received standard care (N=55) N (%)
**Age (Mean, SD)**	74.8 (9.0)	72.7 (9.9)
**Male**	30 (47.6)	14 (25.5)
**Number of Hospitalizations in Past 6 Months (Mean, SD)**	0.63 (0.83)	0.65 (1.2)
**Length of Stay of Hospitalization**		
**≤ 7 days**	25 (39.68)	49 (89.1)
**> 7 days**	38 (60.32)	6 (10.9)
**COPD Severity***		
**Mild or Moderate (FEV1 ≥50% predicted)**	16 (25.4)	15 (27.3)
**Severe or Very Severe (FEV1 less than 50% predicted)**	35 (55.6)	20 (36.4)
**Missing/Unknown**	12 (19.1)	20 (36.4)
**Patient-reported Smoking Status - Yes**	11 (17.5)	38 (69.1)
**Home Oxygen Use**	21 (33.3)	22 (40.0)
**Coronary Artery Disease**	17 (27.0)	10 (18.2)
**eGFR 30 – 60 mL/min**	12 (19.1)	13 (23.6)
**eGFR < 30 mL/min**	3 (4.8)	5 (9.09)
**Hypertension**	38 (60.3)	31 (56.4)
**Heart Failure**	12 (19.1)	11 (20.0)
**Dementia or Cognitive Impairment**	5 (7.9)	17 (30.9)
**Diabetes Mellitus**	18 (28.6)	12 (21.8)
**Atrial Fibrillation or Aflutter**	10 (15.9)	12 (21.8)

**Asthma**	8 (12.7)	7 (12.7)
**Body Mass Index Category***		
**Underweight**	6 (9.5)	6 (10.9)
**Normal**	18 (28.6)	22 (40.0)
**Overweight**	18 (28.6)	7 (12.7)
**Obese (Classes I, II, or III)**	19 (30.2)	20 (36.4)
**Missing/Unknown**	2 (3.2)	0 (0.00)
**Emergency Department Visit within 30 Days (Outcome)**	13 (20.6)	9 (16.4)
**Rehospitalization / Readmission within 30 Days (Outcome)**	8 (12.7)	4 (7.3)

COPD – chronic obstructive pulmonary disease, eGFR – estimated glomerular filtration rate, FEV1 – forced expiratory volume in one second.
*Not included in the propensity score model due to >5% patients missing information.

Makari J, et al. Hospital pharmacist discharge care is independently associated with reduced risk of readmissions for patients with chronic obstructive pulmonary disease: A propensity-matched cohort study. Can Pharm J (Ott) 2022;155. DOI: 10.1177/17151635211061141.
